# Mindful Eating and Current Glycemic Control in Patients With Type 2 Diabetes

**DOI:** 10.7759/cureus.57198

**Published:** 2024-03-29

**Authors:** Ayşe Naciye Erbakan, Muzeyyen Arslan Bahadir, Ozlem Gonen, Fatos Nimet Kaya

**Affiliations:** 1 Department of Internal Medicine, Göztepe Prof. Dr. Süleyman Yalçın Şehir Hastanesi, Medeniyet University, Istanbul, TUR; 2 Department of Internal Medicine, Göztepe Prof. Dr. Suleyman Yalcin City Hospital, Medeniyet University, Istanbul, TUR

**Keywords:** mindful eating, body mass index, glycemic control, type 2 diabetes mellitus (type 2 dm), mindfulness

## Abstract

Objective

Lifestyle adjustments are essential in the management of type 2 diabetes mellitus (T2DM). Mindful eating involves being more attentive to and aware of meals. This study aimed to investigate the relationship between mindful eating and glycemic control, as well as body mass index (BMI), in people with T2DM.

Materials and methods

This cross-sectional study included 448 participants who had been diagnosed with T2DM for at least six months. The participants were categorized into three groups based on their HbA1c levels. The Turkish adaptation of the Mindful Eating Questionnaire (MEQ-30) was employed to assess levels of mindful eating behavior. Obesity was defined as a BMI ≥ 30. Anthropometric measurements, laboratory tests, and questionnaire responses were also collected.

Results

Participants with well-controlled diabetes (HbA1c ≤7%) demonstrated significantly higher scores on the MEQ-30 and its various subgroups in comparison to those with poorly controlled diabetes (HbA1c >9%). The suboptimal glycemic control groups exhibited noticeable variations in mindful eating behaviors. Moreover, participants with lower BMIs displayed stronger inclinations toward mindful eating. Weak negative correlations were observed between BMI and specific MEQ-30 subgroups. Notably, subgroups such as emotional eating, eating control, eating discipline, and interference demonstrated weak negative correlations with the HbA1c levels.

Conclusion

Higher levels of mindful eating were associated with lower levels of HbA1c and BMI, indicating that incorporating mindful eating practices may present promising advantages for individuals diagnosed with type 2 diabetes, specifically in terms of glycemic control and weight management.

## Introduction

Type 2 diabetes mellitus (T2DM) is a global health challenge that requires a comprehensive understanding of the factors that influence its management. Abdominal obesity and insulin resistance play direct roles in the development of T2DM. Lifestyle modifications, including adopting a healthy diet, losing weight, and engaging in regular exercise, are key to combating abdominal obesity and insulin resistance. [1] These changes not only contribute to glycemic control but also have positive effects on the management of other risk factors such as hypertension and hyperlipidemia. Furthermore, they can potentially delay or prevent complications and, in some cases, can even lead to diabetes regression. [2]

Nutritional interventions are crucial for promoting healthy eating habits and facilitating weight loss. However, maintaining these lifestyle changes and ensuring continuity is challenging. Incorporating daily adjustments into food choices can be difficult for individuals with diabetes. Various dietary approaches advocating restricted carbohydrate intake have been proposed to achieve both weight loss and glycemic control. The challenge lies not only in persuading patients to adopt lifestyle changes and adhere to treatment but also in transforming these changes into long-term habits.

The concept of "mindful eating" or "eating awareness" is pivotal to behavioral change and plays a crucial role in the success of nutritional interventions. Mindful eating is an approach in which individuals engage with their eating habits with deep mindfulness and awareness. It involves a conscious immersion in the sensory realms that include taste, texture, and smell, as well as attentive observation of the internal signals that indicate hunger and satiety. An essential part of mindful eating is cultivating a non-judgmental and empathetic attitude toward one's eating habits in order to create an environment that is conducive to holistic well-being. At the heart of the practice is the conscious regulation of eating pace, which allows for a conscious appreciation of each gastronomic encounter [3]. Mindful eating goes beyond simply focusing on portion sizes, ingredients, and meal timing. It also encompasses understanding the emotional influences on eating behavior, i.e. overcoming challenges such as food cravings and disruptions in eating due to stress, anxiety, or depression, including skipped meals and irregular eating habits. They are all crucial for people with obesity to manage diabetes and overall well-being [4,5].

Studies by Fanning et al. [6] and Dunn et al. [7] have demonstrated a positive association between mindful eating and improved dietary adherence, underscoring the importance of incorporating it into weight management programs. The American Heart Association (AHA) acknowledges the impact of irregular eating habits on cardiometabolic health and advocates for mindful eating habits [8]. It emphasizes the significance of mindful eating for a healthier lifestyle and effective management of cardiometabolic risk factors. Patient adherence to mindfulness practices can be assessed through various tests, including the validated Mindful Eating Questionnaire (MEQ-30) [9-11].

By identifying specific aspects of mindful eating associated with optimal glycemic control, our study aimed to provide valuable insights that could facilitate personalized and innovative approaches to diabetes management. Our goal was to improve scientific understanding and develop tailored interventions for individuals with T2DM.

As there is growing evidence to support the integration of mindful eating into standard nutrition education, this study aimed to investigate the relationship between mindful eating behaviors (assessed by the MEQ-30) and glycemic control (measured by HbA1c) in individuals diagnosed with T2DM for at least six months. Furthermore, the effect of obesity on these associations was also examined.

## Materials and methods

In this cross-sectional study, consecutive individuals who were admitted to the Diabetes Outpatient Clinic of Medeniyet University Göztepe Prof. Dr. Suleyman Yalcin City Hospital, Istanbul, Türkiye, between July 1, 2019, and November 30, 2019, who had been diagnosed with T2DM according to the ADA guidelines for at least six months and who had consented to the study were included as participants. The study protocol was approved by the Istanbul Medeniyet University Goztepe Training and Research Hospital Ethics Committee (approval number: 2019/0295) and the study was conducted in accordance with the guidelines outlined in the Declaration of Helsinki. All participants gave informed consent. Exclusion criteria included individuals with a diagnosis of T2DM for less than six months, uncertainty regarding T2DM diagnosis, and the presence of other medical conditions or medications influencing eating preferences. The clinical trial registration number was NCT06229847.

The primary endpoint was to determine the association between the total score and subgroup scores of mindful eating and glycemic control. The secondary endpoint was to examine the relationship between obesity and the total and subgroup scores of mindful eating.

Study design

**Figure 1:**
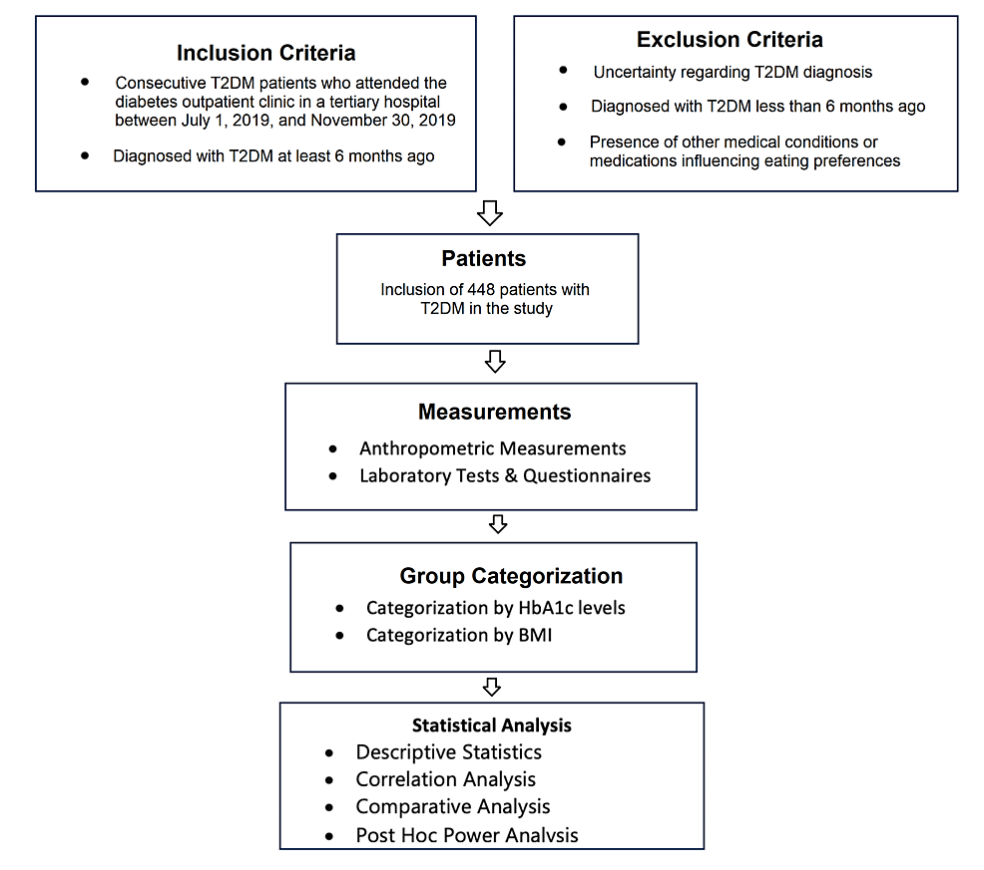
Flowchart of the study T2DM: type 2 diabetes mellitus

A minimum of 300 participants were required to ensure sufficient statistical power. Baseline assessments recorded the duration of diabetes, demographic characteristics, and coexisting diseases for all participants. Figure 1 gives the flow of the study.

Questionnaire

We employed the validated Turkish MEQ-30, consisting of 30 items rated on a scale ranging from 1 (never) to 5 (always). A higher score on this scale indicates a greater level of mindfulness in eating. These items assess seven constructs related to eating behavior and mindfulness, with higher scores indicating a higher level of mindful eating. The seven sub-factors of the MEQ-30 included disinhibition, emotional eating, eating control, mindfulness, eating discipline, conscious nutrition, and interference. Kose et al. established the validity and reliability of the Turkish version of the scale [9]. The scale items were adapted from the MEQ-28, with some modifications made during the translation process. Consequently, two additional questions were included, resulting in a total of 30 questions. Likert-type scales commonly utilize a 5-point scale, as initially proposed by Likert (1932). Therefore, our adapted scale utilizes a five-point Likert scale (1= none, 2= rarely, 3 = sometimes, 4 = often, and 5= always). The items, which were originally organized into five factors in the United States culture, were rearranged into seven factors in Turkish culture. These factors included disinhibition, emotional eating, eating control, awareness, eating discipline, conscious nutrition, and interference, as described below.

Disinhibition

Disinhibition refers to the tendency to eat without considering the consequences. This trait can be assessed by evaluating an individual's ability to stop eating when they feel full. Such assessments provide valuable insights into issues related to self-control, portion control, and time management. This factor consists of five items.

Emotional Eating

Emotional eating is characterized by the consumption of food as a means of addressing emotional hunger and seeking solace or gratification. Examples of emotional eating include turning to unhealthy snacks during times of stress, indulging in chocolate to enhance feelings of happiness, or using food as a coping mechanism during periods of depression. This factor was evaluated by five items.

Eating Control

Eating control pertains to the ability to regulate the pace of eating and maintain control over the process of consumption. It comprised four components.

Awareness

Awareness refers to the act of fully directing one's attention to the taste of food, deliberately excluding other activities or thoughts. This factor encompassed five items.

Eating Discipline

The eating discipline encompasses various aspects of managing meals, such as planning, preparation, portion control, maintaining order, and time management. It was evaluated using four items.

Conscious Nutrition

Conscious nutrition refers to the awareness of one's physical hunger and fullness, understanding of the caloric and nutritional value of foods, knowledge of healthy nutrition principles, and mindfulness of eating habits.

Interference

Interference involves various sensory factors, including olfactory, visual, and auditory cues, as well as the influence of different stimuli such as food or advertisements.

Anthropometric and laboratory measurements

Anthropometric measurements, including waist circumference, height, and body weight, were performed as part of a comprehensive physical assessment. Standard instruments were utilized to measure body weight, waist circumference, and height. Waist circumference was measured according to the guidelines provided by the World Health Organization [12]. The body mass index (BMI) was calculated as the ratio of weight (kg) to height (in square meters).

All participants underwent routine laboratory tests, which included C-peptide and HbA1c concentrations. All blood tests, including fasting C-peptide and HbA1c levels, were conducted after a fasting period of 10-4 hours and analyzed in the central hospital laboratory. Fasting glucose concentrations were determined using the hexokinase technique. Serum creatinine levels were measured using the Kinetic Jaffe technique. Alanine transaminase (ALT) concentrations were determined using an enzymatic technique, specifically without P-5'-P or NADH. Fasting plasma total cholesterol, high-density lipoprotein (HDL) and low-density lipoprotein (LDL) cholesterol, and triglyceride concentrations were quantified using enzymatic methods with the Abbott Architect c16000 and c8000 instruments (Abbott Laboratories, Chicago, Illinois, United States). HbA1c measurements were obtained using a Tosoh HLC-723 G8 variant-mode ion-exchange high-performance liquid chromatography (HPLC) system (Tosoh Corporation, Tokyo, Japan). C-peptide was quantified using an Abbott Architect I2000 autoanalyzer and a chemiluminescence microparticle immunoassay (Abbott Laboratories). The glomerular filtration rate (GFR) was estimated using the Chronic Kidney Disease Epidemiology Collaboration (CKD-EPI) equation, and the presence of proteinuria was evaluated using the protein/creatinine ratio in spot urine.

To facilitate data analysis, patients with T2DM were categorized into three groups based on their HbA1c levels to reflect their glycemic control. Several guidelines recommend an HbA1c level of below or equal to 7% as an indicator of good glycemic control. HbA1c levels greater than 7% signify uncontrolled diabetes, with levels ≥ 9% commonly representing poor control. Thus, the groups were formed as follows: HbA1c ≤7% (well-controlled), HbA1c 7-9% (uncontrolled), and HbA1c > 9% (poorly controlled)..

Statistical analysis

Data analysis was conducted using IBM SPSS Statistics for Windows, Version 23.0 (Released 2015; Armonk, New York, United States). Descriptive statistics were obtained with quantitative variables represented by mean, maximum (max), and minimum (min) values, and qualitative variables represented by percentages. The normality of the distributions was assessed using the Kolmogorov-Smirnov test. For normally distributed variables, means were reported, and intergroup comparisons were performed using Student's t-test. Comparative analysis of qualitative variables was performed using Pearson's chi-square test, with Fisher's exact test used for small sample sizes (≤5). Non-parametric continuous variables were recorded as medians and compared using the Mann-Whitney U test. The interquartile range (IQR) was also provided for variables recorded as medians. For comparisons involving more than two groups, one-way ANOVA was used, followed by post hoc Tukey’s test if the distribution was normal, or Games-Howell test if it was not. Statistical significance was defined as p < 0.05. A post hoc power analysis was conducted to determine the calculated power for the four groups, revealing a value of 83%. The analysis utilized a medium effect size and an alpha error of 0.05.

## Results

The study included 448 participants, 51.3% (n=230) of whom were female. The mean age was 58.4±0.4 years (median 58 years, min=32, max=56, IQR:14.7). The baseline characteristics are summarized in Table 1.

**Table 1:** Demographic characteristics of the participants IQR, interquartile range; HbA1c: glycated haemoglobin

Variables	Data	BMI <30 (N=246)	BMI ≥30 (N=202)	p-value
Age, years, median (IQR)	58 (14.7)	58 (15)	57 (14)	0.179
Gender, male, n (%)	218 (48.7%)	149 (60.6%)	69 (34.2%)	<0.001
Marital status, married, n (%)	393 (87.7%)	216 (87.8%)	177 (87.6%)	0.954
Educational status, n (%)		132 (53.7%)	119 (58.9%)	0.265
Illiterate	23 (5.1%)			
Literate	9 (2.0%)			
Primary School	219 (48.9%)			
Middle School	55 (12.3%)	114 (46.3%)	83 (41.1%)	
High School	92 (20.5%)			
University	50 (11.2%)			
BMI, kg/m^2^, median (IQR)	29.3 (6.9)			
Waist circumference, cm, median (IQR)	100 (16)	94.5 (12)	109 (15)	<0.001
Presence of disease, n (%)	252 (56.3%)	138 (56.1)	114 (56.4)	0.943
Duration of diabetes, years, median (IQR)	10 (10)	10 (10)	10 (9)	0.636
HbA1c, % , median (IQR)	8.5 (4.0)	8.1 (3.9)	8.9 (4.0)	0.195

The mean BMI and waist circumference were 30.0±0.2 kg/m2 (median=29.3, min=16.8, max=49) and 100.7±0.5 cm, respectively. The mean duration of diabetes was 10.8±0.3 years (median=10, min=1, max=35), and the mean HbA1c was 9.1±1.1% (median 8.5, min=5.1, max=18.3).

HbA1c

The participants were categorized into three groups based on their HbA1c levels: Group 1 (n=123) had levels ≤7%, Group 2 (n=120) had levels between 7.01% and 9%, and Group 3 (n=195) had levels >9%. A comparison of the three groups is presented in Table 2.

**Table 2:** Comparison of HbA1c groups in terms of demographics ^1^ Comparison of three groups with each other; ^2^Comparison of Groups 1 and 2 (HbA1c ≤7% with HbA1c 7-9%); ^3^Comparison of Groups 1 and 3 (HbA1c ≤7% with HbA1c >9%); ^4^Comparison of Groups 2 and 3 (HbA1c 7-9% with HbA1c >9%). p-values in bold indicate statistical significance. IQR, interquartile range; HbA1c, glycated haemoglobin

Variables	Group 1 HbA1c ≤7 (N=123)	Group 2 HbA1c 7-9 (N=130)	Group 3 HbA1c >9 (N=195)	p-value^1^	p-value^2^	p-value^3^	p-value^4^
Age, years, median (IQR)	58 (13)	60 (16)	57 (16)	0.08	0.365	0.741	0.07
Gender, male, n (%)	63 (51.2%)	57 (43.8%)	98 (50.3%)	0.423	0.471	0.985	0.494
Marital status, married n (%)	113 (91.9%)	116 (89.2%)	164 (84.1%)	0.100	0.754	0.08	0.366
Educational status, n (%)				0.006	0.02	0.008	0.981
Primary school and below	54 (43.9%)	78 (60.0%)	119 (61.0%)				
Secondary school and above	69 (56.1%)	52 (40.0%)	76 (39.0%)				
BMI, kg/m^2^, median (IQR)	29.3 (7.0)	29.2 (5.9)	29.6 (7.3)	0.850	0.907	0.843	0.994
Waist circumference, cm, median (IQR)	97 (18)	98 (14)	101 (14)	0.01	0.733	0.01	0.101
Presence of disease, n (%)	75 (61.0)	79 (60.8)	98 (50.3)	0.08	0.999	0.145	0.147
Duration of diabetes, years, median (IQR)	9 (10)	10 (9)	10 (10)	0.254	0.216	0.644	0.619

Significant differences were observed between the groups in terms of educational status and waist circumference, with p-values of 0.006 and 0.01, respectively. Group 1, the group with the lowest HbA1c levels, had a higher percentage of participants with higher education. However, no statistically significant difference in educational status was found between groups 2 and 3 (p=0.981). While waist circumference did not differ significantly between groups 1 and 2 (p=0.733), it was significantly lower in Group 1 compared to Group 3 (p=0.01).

MEQ-30 scores**

Scores were computed separately for the seven subgroups of mindful eating as well as for the overall MEQ-30. A comparison among the three groups in terms of all mindful eating subgroups is presented in Table 3.

**Table 3:** Comparison of HbA1c groups in terms of all eating parameters ^1^ Comparison of three groups with each other; ^2^Comparison of groups 1 and 2 (HbA1c ≤7% with HbA1c 7-9%); ^3^Comparison of groups 1 and 3 (HbA1c ≤7% with HbA1c >9%); ^4^Comparison of groups 2 and 3 (HbA1c 7-9% with HbA1c >9%). p-values in bold indicate statistical significance. HbA1c, glycated haemoglobin

Eating parameters	Group 1 HbA1c ≤7% (N=123)	Group 2 HbA1c 7-9% (N=130)	Group 3 HbA1c >9% (N=195)	p-value^1^	p-value^2^	p-value^3^	p-value^4^
Disinhibition	3.8 (1.2)	3.8 (1.2)	3.4 (1.6)	<0.001	0.479	<0.001	0.001
Emotional eating	4.4 (0.8)	4.6 (1.0)	4.2 (1.6)	<0.001	0.983	<0.001	<0.001
Eating control	3.5 (1.0)	3.5 (1.0)	3.2 (1.0)	0.051	0.655	0.04	0.311
Awareness	3.4 (0.6)	3.4 (0.6)	3.4 (0.8)	0.667	0.920	0.888	0.646
Eating discipline	3.2 (1.5)	3.0 (1.0)	3.0 (1.2)	0.01	0.989	0.03	0.03
Conscious nutrition	3.2 (0.8)	3.0 (1.0)	3.0 (0.8)	<0.001	0.02	<0.001	0.360
Interference	4.0 (1.0)	4.0 (1.0)	4.0 (1.5)	0.004	0.959	0.007	0.02
Total mindful eating	3.6 (0.5)	3.5 (0.4)	3.4 (0.6)	<0.001	0.421	<0.001	<0.001

Significant differences were found in the scores for disinhibition (p<0.001), emotional eating (p<0.001), eating discipline (p=0.01), conscious nutrition (p<0.001), interference (p=0.004), and total mindful eating (p<0.001) between the groups. Eating control demonstrated a statistically significant difference that approached significance (p=0.05) among the three groups. However, there were no significant differences between groups in terms of awareness (p=0.667).

The only subgroup that displayed a significant difference was conscious nutrition between groups 1 and 2. This study revealed significant differences in all subgroups except for "awareness" when comparing Group 1 with HbA1c < 7% (representing good glycemic control) and Group 3 with HbA1c > 9% (indicating poor control). In this comparison, Group 3 exhibited lower MEQ-30 scores. For groups 2 and 3, significant differences were observed in all subgroups except for eating control, awareness, and conscious nutrition, where Group 2 presented higher MEQ-30 scores.

When examining the subgroups individually, the disinhibition subgroup showed no differences between groups 1 and 2 (p=0.479), whereas the score was significantly lower in the group with poorly controlled diabetes than in groups 1 and 2 (p<0.001 and p=0.001, respectively). In terms of emotional eating, the scores were comparable in groups 1 and 2 (p=0.983), while the group with poorly controlled diabetes had lower scores than groups 1 and 2 (p<0.001 and p<0.001, respectively). Regarding eating control, the difference was significant only between groups 1 and 3 (p=0.04). For eating discipline, the scores for groups 1 and 2 were similar (p=0.989), but Group 3 differed significantly from both groups 1 and 2 (p=0.03, p=0.03, respectively). Mindfulness scores were higher in the well-controlled diabetes group than in Group 2 (p=0.02) and Group 3 (p<0.001), but there was no difference between groups 2 and 3 (p=0.360). In terms of interference, the scores were similar in groups 1 and 2 (p=0.959), whereas significant differences were observed between Group 3 and groups 1 and 2 (p=0.007 and p=0.02, respectively). For the total mindful eating score, groups 1 and 2 were similar (p=0.421), whereas the group with poorly controlled diabetes had lower scores than groups 1 and 2 (p<0.001 and p<0.001, respectively).

BMI

The participants were then divided into two groups based on their BMIs: participants with a BMI <30 (n=246) and participants with a BMI ≥ 30 (n=202). The demographic characteristics and comparison of these two groups are outlined in Tables 1, 4.

**Table 4:** Comparison of BMI groups in terms of mindful eating and its subgroups p-values in bold indicate statistical significance.

Eating parameters	BMI <30 (N=246)	BMI ≥30 (N=202)	p-value
Disinhibition	3.8 (1.2)	3.5 (1.6)	0.02
Emotional eating	4.4 (0.8)	4.2 (1.4)	0.04
Eating control	3.5 (1.0)	3.2 (1.0)	0.067
Awareness	3.4 (0.6)	3.4 (0.6)	0.313
Eating discipline	3.0 (1.2)	3.0 (1.2)	0.01
Conscious nutrition	3.0 (0.8)	3.0 (0.8)	0.127
Interference	4.0 (1.0)	4.0 (1.5)	0.02
Total mindful eating	3.5 (0.5)	3.4 (0.6)	<0.001

Participants with a BMI ≥ 30 were more likely to be female. The correlations between BMI, HbA1c, and duration of diabetes in the mindful eating subgroups are shown in Table 5.

**Table 5:** Correlation analysis between BMI, HBA1c, and duration of diabetes and eating parameters p-values in bold indicate statistical significance. Rho, spearman correlation coefficient; HbA1c, glycated haemoglobin

	BMI		HbA1c		Duration of diabetes	
Eating parameters	Rho	p-value	Rho	p-value	Rho	p-value
Disinhibition	-0.127	0.007	-0.253	<0.001	0.025	0.592
Emotional eating	-0.159	0.001	-0.171	<0.001	0.073	0.123
Eating control	-0.123	0.009	-0.103	0.03	0.004	0.924
Awareness	-0.072	0.130	0.004	0.940	-0.023	0.624
Eating discipline	-0.132	0.005	-0.166	<0.001	0.145	0.002
Conscious nutrition	-0.123	0.009	-0.206	<0.001	-0.016	0.735
Interference	-0.113	0.01	-0.155	0.001	-0.012	0.793
Total mindful eating score	-0.207	<0.001	-0.261	<0.001	0.049	0.299

A significant, yet weak, negative correlation was observed between BMI and specific subgroups of mindful eating, including disinhibition, emotional eating, eating control, eating discipline, mindfulness, and interference. Similarly, the correlation between BMI and overall mindful eating score was also weak but significant in the reverse direction. No significant correlations were found between other subgroups of mindful eating and BMI.

Regarding HbA1c, weak but significant negative correlations were found with the subgroups of mindful eating such as emotional eating, eating control, eating discipline, and interference. The subgroups of disinhibition, mindfulness, and overall mindful eating scores were also negatively correlated with HbA1c levels, albeit weakly. No significant correlations were observed between other eating parameters and HbA1c levels.

Only a very weak significant correlation was found between the duration of diabetes and the eating discipline. No other correlations were observed between the duration of diabetes and other eating parameters.

## Discussion

The results of this study revealed that people with T2DM with better glycemic control had higher scores on the MEQ-30 and its subgroups, indicating a higher mindful eating status. In addition to examining the association between HbA1c and mindful eating scores, this study sets itself apart from previous research by categorizing diabetes patients based on their glycemic control and assessing the discrepancy in mindful eating scores between individuals with adequately and inadequately controlled diabetes.

Participants with elevated HbA1c levels, indicating poor glycemic control (HbA1c > 9%), exhibited lower scores in conscious nutrition, disinhibition, emotional eating, eating discipline, interference, and the overall MEQ-30 score than those with better glycemic control (HbA1c < 7%). These findings imply that individuals with T2DM may enhance their glycemic control by adopting a mindful eating approach. The results of this study suggest that mindful eating may be beneficial for individuals with T2DM in improving glycemic control. The teachings associated with the practice of mindful eating can be recommended. This has the potential to increase the impact of personalized nutrition plans formulated by dietitians, as well as the effectiveness of nutritional counseling offered to individuals regarding weight management and improving eating habits [13 ]. In a study conducted by Miller et al., both mindful eating and diabetes self-management education (DMSE) were found to be effective interventions for enhancing eating regulation and dietary patterns, and reducing symptoms of depression and anxiety in adults with T2DM [14]. DMSE is recommended in diabetes guidelines as a fundamental therapy [15].

In a recent study, the incorporation of mindfulness-based interventions alongside standard care resulted in significant improvements in participants' HbA1c and fasting glucose levels. [16] Another study conducted by Loucks et al. on a cohort of adults revealed that individuals with high levels of dispositional mindfulness were more likely to maintain normal plasma glucose levels [17]. This association was partially mediated by a reduced likelihood of obesity and enhanced perception of personal control.

Distinct differences were identified among the various subgroups when comparing groups 2 and 3. Group 2 exhibited higher MEQ-30 scores for disinhibition, emotional eating, eating discipline, mindfulness, and interference. Nevertheless, no significant differences were observed in terms of eating control, awareness, or conscious nutrition. These findings indicate that even within suboptimal glycemic control ranges, variations in mindful eating behaviors may exist, underscoring the need for personalized interventions. Prior research has shown a link between the consumption of unhealthy foods such as sweets, sweetened soft drinks, and unhealthy snacks, and emotional and external eating patterns [18,19]. Consequently, fostering a heightened sense of eating awareness could be considered an advantageous dietary approach with a positive impact on food consumption, thereby promoting health and disease prevention [20].

Further examination of the data involved categorizing participants into groups based on body mass index (BMI). This analysis revealed weak yet significant negative associations between BMI and specific subgroups of mindful eating. Specifically, participants with higher BMIs exhibited lower disinhibition, emotional eating, eating control, eating discipline, mindfulness, interference, and overall mindful eating scores. These findings suggest that individuals with lower BMIs may possess a more mindful approach to eating, which could potentially contribute to improved metabolic outcomes. These findings are consistent with several studies demonstrating a correlation between obesity and decreased levels of mindful eating among individuals [21-23]. In Özkan et al.'s study, although the study population did not specifically consist of individuals with diabetes, higher MEQ-30 scores were associated with lower anthropometric measurements [24].

In our study, there were modest yet noteworthy negative associations between HbA1c levels and emotional eating, eating control, eating discipline, and interference. Similarly, the disinhibition, mindfulness, and overall scores for mindful eating were slightly negatively correlated. These findings suggest that certain facets of mindful eating may affect glycemic control. Therefore, it is imperative to consider these elements in the management of diabetes to regulate blood glucose levels effectively. Ni et al. conducted a study that investigated the influence of mindfulness-based interventions (MBI) on glycemic control [25]. Despite identifying inconsistencies in the findings, they ultimately determined that MBI seemed to have positive effects on HbA1c levels in individuals with diabetes. The review encompassed an analysis of eight studies, with mindful eating being included in only one study, potentially contributing to the observed variations.

Mindful eating behaviors were initially developed for individuals with binge eating disorders. The study findings demonstrate a significant improvement in dysregulated eating among individuals with diabetes [14]. Considering the evidence regarding the impact of mindfulness on weight, HbA1c levels, and eating-related outcomes, incorporating principles of mindful eating into diabetes management and self-care interventions, such as DSME, may yield beneficial results [26]. Current knowledge indicates that individuals with dysglycemia often lack awareness of or practice in making healthy food choices [27,28]. Research suggests that both stress- and nutrition-based mindfulness approaches can aid in glycemia and weight management [14]. However, existing studies mainly focus on individuals with diabetes as a whole group in regard to glycemic control. Our approach is different because it compares individuals with varying degrees of glycemic control following a diagnosis of T2DM who have previously received at least six months of pragmatic diabetes therapy with lifestyle change counseling. Further research into the impact of mindful eating combined with lifestyle changes in patients who have not met treatment goals could improve the success of diabetes management.

The study benefits from a robust sample size of participants diagnosed with T2DM, which increases the statistical power of the results and improves the generalizability of the results to the target population. Additionally, the use of the national adaptation of the MEQ-30 enabled a comprehensive assessment of mindful eating behavior in a more reliable way.

It is important to acknowledge the limitations of this study, including its cross-sectional design, which prevents the establishment of causation. Furthermore, reliance on self-reported data and the subjective nature of mindful eating assessment tools may introduce bias. Conducting longitudinal studies in the future and incorporating interventions with mindfulness-based approaches may offer greater insight into causal relationships and potential therapeutic applications for managing T2DM.

The results of this study have important implications for the management of T2DM. First, the observed association between higher levels of mindful eating and improved glycemic control suggests that incorporating mindful eating practices into lifestyle interventions for individuals with T2DM could lead to favorable outcomes in glycemic control. This emphasizes the potential of mindful eating as a complementary approach to traditional dietary interventions in T2DM management programs. Second, the significant correlations between mindful eating behaviors and BMI suggest that promoting mindful eating may also have benefits for weight management in individuals with T2DM, potentially contributing to overall health improvement and disease prevention. Finally, the identification of specific subgroups of mindful eating behaviors associated with optimal glycemic control provides valuable insights for the development of personalized interventions tailored to the unique needs of patients with T2DM. Overall, these findings underscore the importance of integrating mindful eating habits into diabetes treatment strategies to optimize clinical outcomes and improve the quality of life for people with T2DM.

## Conclusions

Our study provides valuable insights into the relationship between mindful eating, glycemic control, and BMI in individuals diagnosed with T2DM. The differences in mindful eating categories observed among the HbA1c-defined groups underscore the potential significance of mindful eating in blood glucose management. Tailored interventions focusing on specific aspects of mindful eating could offer promising prospects for improving the metabolic outcomes in this population. Further studies are warranted to explore the long-term effects of mindfulness-based interventions on glycemic control and overall well-being in individuals with T2DM.
